# Beyond data gaps: how prior knowledge in the Gulf of California drives spatial biases in whale survey efforts

**DOI:** 10.7717/peerj.21685

**Published:** 2026-09-10

**Authors:** Omar García-Castañeda, Jorge M. Lobo, Jorge Urbán R., Lili Pelayo-González, Alejandro Gómez-Gallardo U.

**Affiliations:** 1 Facultad de Ciencias, Universidad Nacional Autónoma de México, Ciudad de México, México; 2 Departamento Académico de Ciencias Marinas y Costeras, Universidad Autónoma de Baja California Sur, La Paz, Baja California Sur, México; 3 Department of Biogeography and Global Change, Museo Nacional de Ciencias Naturales (CSIC), Madrid, Spain

**Keywords:** Survey bias, Cetacean data, Knowledge-limited inventory, Environmental drivers, Whale monitoring

## Abstract

We analysed whale survey effort conducted by multiple research groups to evaluate whether sampling-limited or knowledge-limited inventory processes better explain observed gaps in whale records. Using data from the Gulf of California, survey effort was quantified as the number of navigation routes and travelled kilometres crossing grid cells at five spatial resolutions. Its spatial distribution was examined in relation to environmental and geographic variables using Generalised Linear Models. Results indicate that survey effort was highly concentrated across all spatial resolutions, with fewer than 10% of grid cells accounting for the highest levels of sampling. Environmental predictors explained only a limited proportion of this spatial variation. However, shallow bathymetry, colder waters, and higher productivity were consistently associated with greater survey effort. Analyses of residuals and spatial autocorrelation revealed systematic underestimation of effort in a small number of heavily surveyed areas, indicating sampling intensities far exceeding those expected based solely on environmental conditions. These over-surveyed areas coincided with independently identified hotspots of whale occurrence, suggesting that survey effort has been preferentially directed towards locations already known to support high whale densities. Biases affecting biodiversity databases are commonly interpreted as resulting from insufficient sampling; however, they may also reflect the influence of prior knowledge guiding where data are collected. Our results therefore support a knowledge-limited inventory scenario, in which accumulated experience and expectations shape survey design and reinforce existing spatial biases.

## Introduction

Biodiversity databases compile information on a wide range of biological attributes, including species occurrences, population counts or community composition; however, they are almost invariably affected by multiple sources of bias and incompleteness, which may be taxonomic, geographical, or environmental in nature (Daru & Rodriguez, 2023; García-Roselló, González-Dacosta & Lobo, 2023). These shortcomings largely arise from the uneven distribution of taxonomic and faunistic research effort, driven by differences in scientific interest, availability of resources, and long-standing preferences for particular organisms, regions, or habitat types (*e.g.*, Embling, Walters & Dolman, 2015; Tyne et al., 2016; Tang, Clark & Gelfand, 2021; Davis, Guralnick & Zipkin, 2023). When these data are mapped, such disparities translate into areas with few or insufficient records, which are typically regarded as poorly surveyed and identified as “data gaps” requiring additional sampling effort. This situation is widespread across most taxonomic groups and geographical regions, and is particularly common in biodiversity-rich areas and during the early phases of inventory development. Under these circumstances, it is advisable to assess the environmental and spatial range encompassed by well-surveyed localities (Austin & Heyligers, 1989; Rocchini et al., 2011) in order to identify the minimum number and spatial distribution of additional sites that should be sampled to adequately represent the environmental and geographical variability of the region under study. Such an approach facilitates the construction of datasets that are more suitable for reliable biodiversity modelling and prediction (Hortal & Lobo, 2005; Hortal, Lobo & Jiménez-Valverde, 2007; Guralnick, Hill & Lane, 2007).

Alternatively, gaps and biases in biological information may also arise through a less frequently considered mechanism that effectively reverses the apparent causal relationship between the biological attribute of interest and sampling effort. Under this alternative scenario, low observed data values in a locality are not the consequence of insufficient sampling (hereafter, sampling-limited inventories). Instead, limited sampling occurs because the area has previously been identified as biologically uninformative or lacking naturalistic interest (knowledge-limited inventories). Under this scenario, the existence of published or unpublished prior knowledge may discourage further sampling in certain areas, thereby reinforcing their perceived biological irrelevance (Dennis & Thomas, 2000). This process is likely to emerge once a region has surpassed a certain threshold of inventory completeness (Sastre & Lobo, 2009), particularly in areas that have been intensively surveyed over time. As sampling effort becomes increasingly selective, areas perceived as offering limited future returns tend to be avoided, while collection activities concentrate progressively on sites known to be productive, accessible, or logistically convenient for obtaining records of target organisms (Haque et al., 2017). As a result, spatial biases in biodiversity databases may reflect not only limitations in survey coverage, but also the cumulative effects of historical knowledge and sampling decisions. Discriminating whether the available biological information in a region reflects a sampling-limited or a knowledge-limited process remains an unresolved and unstudied issue. Here, we propose that under a sampling-limited scenario, localities with little or no information could potentially harbour either low or high values of the biological attribute of interest. In contrast, under a knowledge-limited scenario, understudied localities are expected to contain low values of the biological attribute, and sites with high values should attract a disproportionate number of observations and records.

Cetacean data provide a particularly suitable framework for exploring these alternative scenarios, as they frequently exhibit strong biases in inventory completeness due to the extensive geographical ranges of many species and the methodological challenges associated with detecting individuals (Redfern et al., 2006; Higby, Stafford & Bertulli, 2012; Derville et al., 2018). As a consequence, survey effort is often highly heterogeneous in space, strongly influencing the opportunistic nature of cetacean records and the structure of available databases. These biases can, in turn, affect the identification of areas that maximize the likelihood of detecting cetaceans and obtaining suitable observations (Corkeron et al., 2011). In this study, we analysed compiled whale sighting data from the Gulf of California, a highly biodiverse region considered one of the areas with the greatest cetacean diversity worldwide (Urbán et al., 2005). At least six species of balaenopterid whales are known to occur in the region (Niño-Torres, Urbán-Ramírez & Vidal, 2011), exhibiting contrasting ecological requirements and life-history strategies. For example, humpback whales migrate to the Gulf primarily for breeding and calf-rearing, with feeding rarely observed (Calambokidis et al., 2001), whereas blue whales, despite also using the region for reproduction, must continue feeding due to their large energetic demands (Branch et al., 2007; Sears et al., 2013). In addition, resident populations of fin whales and Bryde’s whales complete their entire life cycles within the Gulf of California (Bérubé et al., 2002; Viloria-Gómora et al., 2021). The long-term presence of multiple whale species has promoted the development of several research programmes recording sightings since the 1980s (Aguayo et al., 1986), each operating with distinct objectives, priorities and logistic constraints. This raises the possibility that whale databases in the region may reflect not only biological patterns, but also historically structured sampling decisions.

Here, we move beyond the conventional interpretation of data gaps as the outcome of insufficient sampling and explicitly test an alternative, knowledge-limited inventory scenario, in which accumulated experience and expectations influence where survey effort is allocated. Under a purely sampling-limited process, areas with low survey effort would not be expected to show systematic deviations from environmentally driven expectations. In contrast, a knowledge-limited process predicts a disproportionate concentration of survey effort in locations already known to support high whale occurrence, resulting in spatial patterns of effort that cannot be explained by environmental conditions alone. Accordingly, we analyse whale survey routes and travelled kilometres conducted by multiple research groups in the Gulf of California to (i) characterise the spatial distribution of survey effort, (ii) identify locations where sampling is disproportionately concentrated or scarce, (iii) assess the environmental variables associated with survey effort across spatial scales, and (iv) determine whether the observed patterns are more consistent with a sampling-limited or a knowledge-limited inventory process. By addressing these objectives, this study aims to contribute to the design of improved survey strategies for cetaceans in the Gulf of California and to advance the understanding of how sampling efforts shape the reliability of spatial predictions of whale distributions.

## Materials and Methods

### Study region

The Gulf of California is a semi-enclosed sea located between the Baja California Peninsula and north-western Mexico. It is characterized by distinctive oceanographic conditions that are partially isolated from the Pacific Ocean. The region experiences a strong monsoonal regime, resulting in high biological productivity during winter and spring, followed by more oligotrophic conditions in summer and autumn (Alvarez-Borrego & Lara-Lara, 1991). Nevertheless, certain areas remain productive throughout the year owing to persistent upwelling or intense vertical mixing driven by strong tidal regimes or specific geomorphological features. These processes support a rich and diverse macrofaunal community (Brusca et al., 2005).

### Data sources and survey effort

A map of whale surveys in the Gulf of California was developed based on the navigation routes followed by tracking and research teams, together with the calculation of the kilometres travelled during surveys. Whale surveys are typically conducted from small outboard vessels (7–8 m in length), during which researchers activate portable Global Positioning System (GPS) devices at the start of each navigation. Whale positions are recorded at the time of sighting, and the complete navigation route is downloaded from the GPS unit at the end of each survey. Although navigation may be partially directed towards areas where whales are expected to occur, survey effort is effectively distributed along the entire route, as whales are highly mobile and spend most of their time underwater. Importantly, GPS recording is stopped whenever survey activity ceases. Consequently, all recorded navigation segments represent active search effort under broadly comparable observational conditions, and we therefore consider navigation routes and travelled kilometres to be reliable proxies of spatial survey effort.

**Figure 1:**
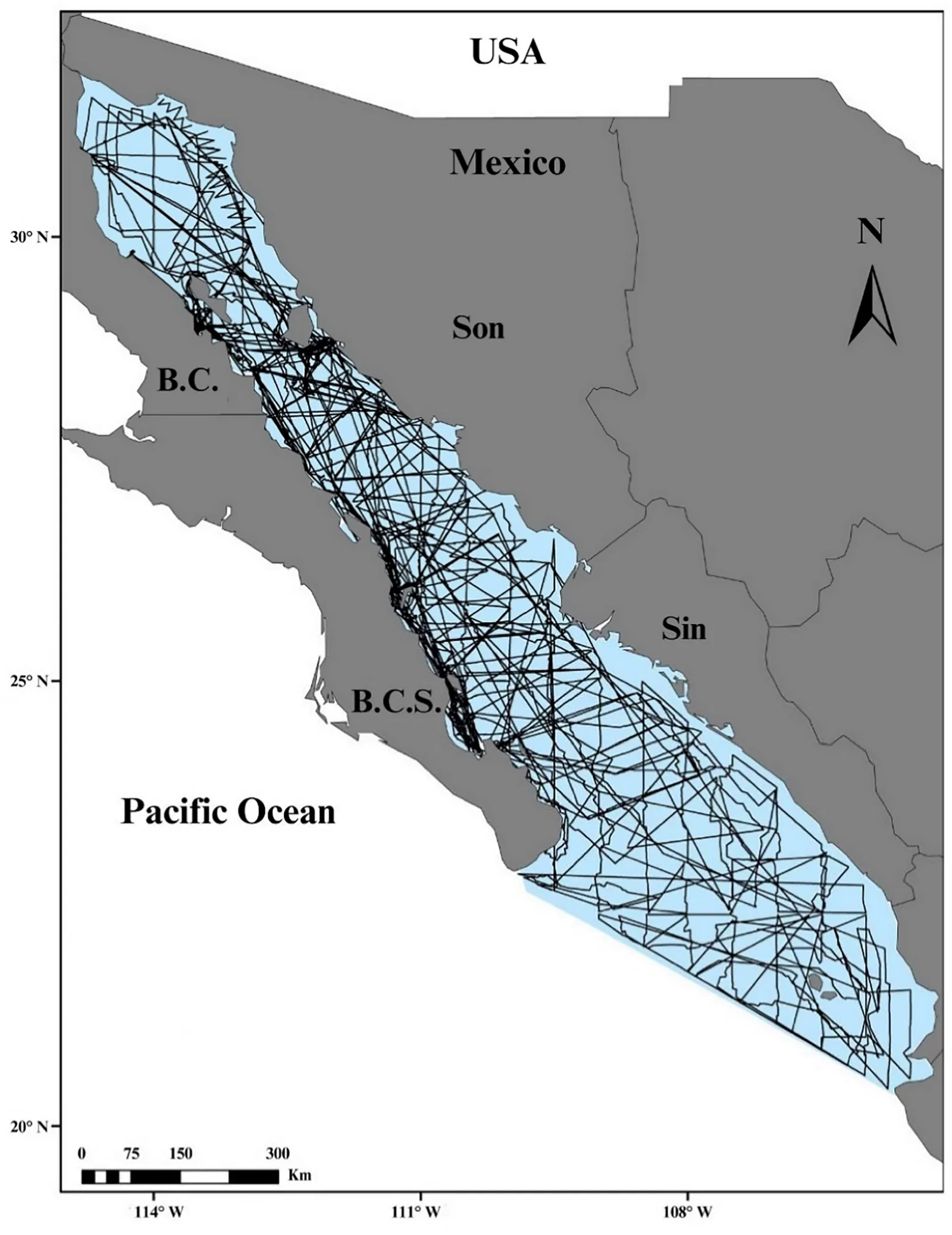
Map of navigation routes. Map illustrating the navigation routes followed by tracking and research teams in the Gulf of California between 1987 and 2018. Acronyms: Sonora (Son), Sinaloa (Sin), Baja California (B.C.), Baja California Sur (B.C.S.).

The primary data source was the Programa de Investigación de Mamíferos Marinos of the Universidad Autónoma de Baja California Sur (PRIMMA-UABCS; https://sites.google.com/uabcs.mx/primma/). This database compiles navigation routes and cetacean sightings collected between 1987 and 2018. To improve spatial and temporal coverage, these data were complemented with information from additional institutions, including the Centro Interdisciplinario de Ciencias Marinas (CICIMAR-IPN), the Centro de Investigación Científica y Estudios Superiores de Ensenada (CICESE), the Prescott College Research Center, and the Programa de Observación de Cetáceos (PROCETUS). Together, these sources provide a comprehensive representation of historical whale survey effort across the Gulf of California (Fig. 1).

### Spatial framework and grain size

**Figure 2:**
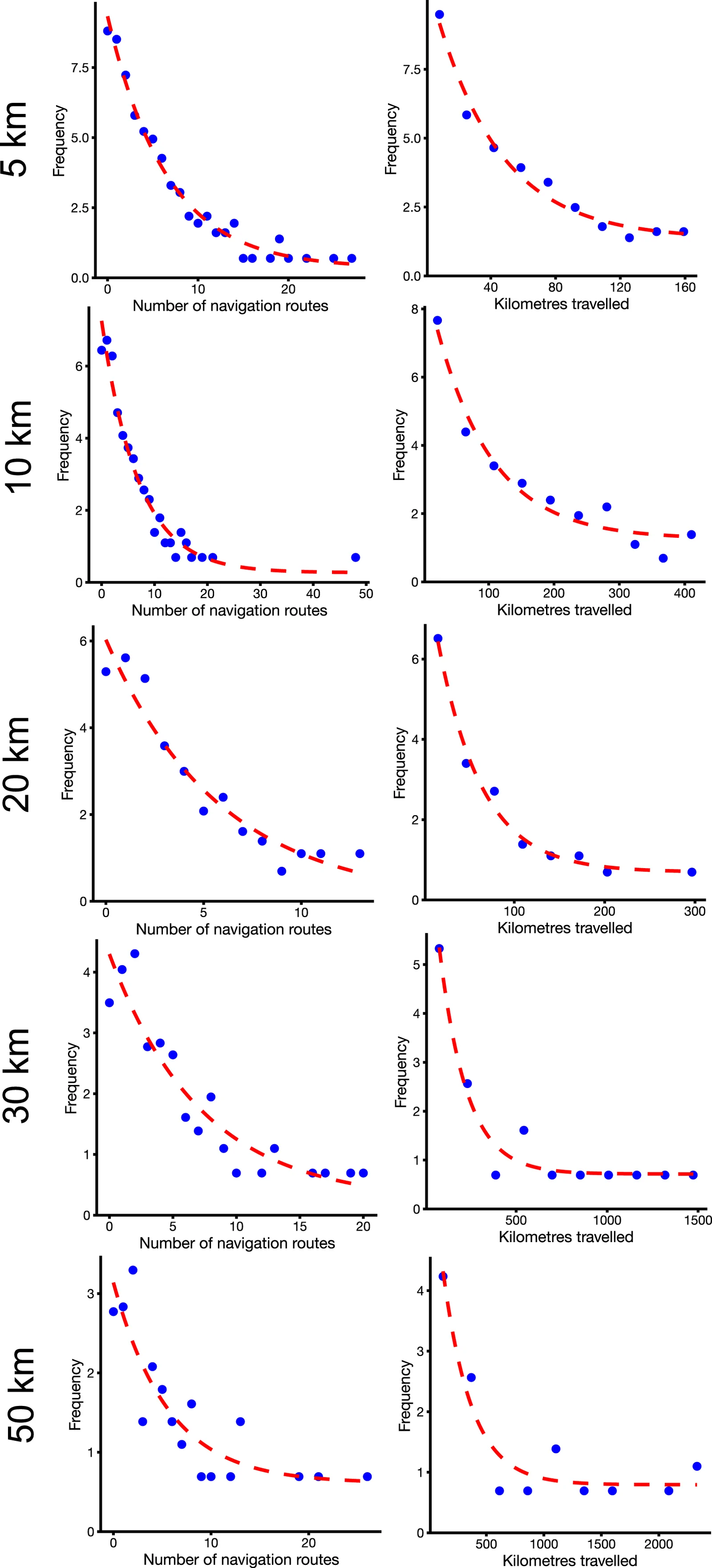
Frequency distribution of grid cells. Frequency distribution (expressed as ln + 1) of grid cells according to the number of navigation routes crossing each cell and the kilometres travelled (x-axes). Distributions were calculated for each one of the five spatial resolutions considered. Observed values were fitted using negative exponential functions (red dashed lines). For travelled kilometres, ten bins were defined independently for each spatial resolution according to the minimum and maximum valued recorded per cell.

To quantify survey effort, the study area was divided into grid cells at five spatial resolutions: 5 × 5 km (*n* = 13,787 cells), 10 × 10 km (*n* = 2,289), 20 × 20 km (*n* = 726), 30 × 30 km (*n* = 229) and 50 × 50 km (*n* = 91). Survey effort was quantified both as the number of navigation routes crossing each cell (ROCELL) and as the total of kilometres travelled by these routes within each cell (KMCELL). When a route was repeated multiple times, each occurrence and travelled distance were counted separately, even if the spatial trajectory was identical. Frequency distributions of survey effort surrogates were examined for each spatial resolution. To identify spatial bias in survey effort, cells with numbers of navigations routes and travelled kilometres equal to or greater than the values corresponding to the 90th and 95th percentiles were classified as relatively well-surveyed. These percentile-based thresholds were intended to provide les conservative (90th percentile) and more conservative (95th percentile) criteria for identifying cells with comparatively high survey effort. To illustrate the decline in the number of cells as survey effort increases, frequency distributions were fitted to a negative exponential function using CurveExpert 1.4 software (www.curveexpert.net). This approach enabled both visual and quantitative assessment of how survey effort becomes progressively concentrated at finer spatial resolutions (see Fig. 2). In addition, differences in explanatory power among spatial resolutions were explored to assess scale-dependent bias. ROCELL reflects the spatial concentration of navigation trajectories, whereas KMCELL mainly reflects traffic intensity. Although Pearson correlation coefficients between ROCELL and KMCELL values were consistently positive and statistically significant (*p* < 0.001), ranging from 0.631 to 0.704, all analyses were conducted using both survey-effort surrogates to confirm the robustness of the results obtained.

### Environmental and geographic predictors

Geographical and environmental variables potentially influencing whale survey effort were compiled from multiple sources (Table 1). Environmental predictors were incorporated as multi-annual averages in order to characterise the dominant spatial environmental gradients of the Gulf of California rather than interannual environmental variability. Several of these variables have previously been shown to correlate with cetacean distributions (Davis, Guralnick & Zipkin, 2023; Pardo et al., 2013; Redfern et al., 2017; Becker et al., 2019; Chavez-Rosales et al., 2022). Digital cartography was obtained from NASA’s OceanColor Web platform (https://oceancolor.gsfc.nasa.gov/), from which monthly mean values of sea surface temperature (SST), particulate organic carbon (POC), and chlorophyll a concentration (CHLA) were extracted and averaged for the period June 2002 to April 2018. Additionally environmental variables able to explain the distribution of aquatic species (Tyberghein et al., 2012; Jueterbock et al., 2013; Stuart-Smith et al., 2013; Assis et al., 2018) were obtained from the Bio-ORACLE platform (http://www.bio-oracle.org/) as average values for the same period. These variables included calcite concentration (CAL), diffuse attenuation (DA), dissolved molecular oxygen (DMO), nitrate (NIT), pH (PH), phosphate (PHO), phytoplankton concentration (PHY), primary productivity (PP), salinity (SAL), and cloud cover (CC). Bathymetry (BAT) and slope (SLO) were derived from the General Bathymetric Chart of the Oceans (GEBCO; https://www.gebco.net/). Distance to the nearest populated locality (DP) was obtained from digital cartography provided by the Instituto Nacional de Estadística y Geografía de México (INEGI; https://www.inegi.org.mx/). All seventeen variables were resampled to each of the five resolutions using the “average” resampling technique (mean value of all pixels within each grid cell) to ensure comparability across scales. This process was carried out using ArcGIS software version 10.5.1.

**Table 1:** Dependent variables used as surrogates of survey effort. Number of navigation routes per grid cell (ROCELL) and total kilometres of routes crossing each cell (KMCELL), together with the selected explanatory environmental and geographic variables retained after iterative removal of predictors with variance inflation factor (VIF) values greater than 10. Acronyms, units, data sources, and original spatial resolution (expressed in kilometres according to the mean latitude of the Gulf of California) are provided for each variable. Explanatory variables derived from the OceanColor platform were calculated as annual means by averaging monthly values over the period 2002–2018.

	Acronym	Units	Source	Resolution
*Dependent variables*				
Number of routes crossing a cell	ROCELL	n cell^−1^	PRIMMA-UABCS	–
Total kilometres of the routes within a cell	KMCELL	km cell^−1^	PRIMMA-UABCS	–
*Explanatory variables*				
Bathymetry	BAT	m	GEBCO	∼500 m
Chlorophyll *a*	CHLA	mg.m^−3^	OceanColor	∼4 km
Dissolved molecular oxygen	DMO	mol.m^−3^	Bio-ORACLE	∼5 km
Distance to population	DP	m	Processed data from INEGI^*a*^	–
Particulate organic carbon	POC	mg.m^−3^	OceanColor	∼4 km
pH	PH	–	Bio-ORACLE	∼5 km
Phytoplankton	PHY	umol.m^−3^	Bio-ORACLE	∼5 km
Primary productivity	PP	g.m^-3^.day^−1^	Bio-ORACLE	∼5 km
Sea surface temperature	SST	°C	OceanColor	∼4 km
Slope	SLO	%	Processed data from GEBCO^*b*^	–

**Note:**

^a^https://www.inegi.org.mx/. ^b^https://www.gebco.net/.

### Variable selection and collinearity control

Collinearity among environmental predictors can compromise the estimation of regression coefficients and obscure the effects of individual variables (Graham, 2003). To address this issue, variance inflation factors (VIFs) were calculated at all considered spatial resolutions to identify predictors exhibiting excessive multicollinearity (Booth, Niccolucci & Schuster, 1993). Variable selection was performed using ModestR v4.0 (García-Roselló et al., 2013), iteratively removing variables with VIF values greater than 10 (Booth, Niccolucci & Schuster, 1993; Dormann et al., 2013). Only variables showing acceptable collinearity levels across all spatial resolutions were retained for subsequent analyses. As a result, seven explanatory variables were excluded (Table 1). The remaining predictors retained for analysis were BAT, CHLA, DMO, DP, POC, PH, PHY, PP, SST, and SLO.

### Statistical analysis

Generalized Linear Models (GLMs) were used to assess the influence of the selected explanatory variables on spatial variation in both survey-effort surrogates. Survey-effort variables were modelled assuming a Poisson error distribution with a logarithmic link function (Crawley, 1993). Models diagnostics indicated no substantial overdispersion. Type III sums of squares were used to evaluate the partial effect of each predictor while controlling for the influence of the remaining variables. All predictors were standardised (mean = 0, SD = 1) prior to analysis in order to eliminate the effects of differences in measurement scale. Model goodness-of-fit was assessed using deviance (Dev), and parameter significance was evaluated using Wald statistics, with particular attention paid to predictors that were statistically significant for both response variables used as surrogates of survey effort. To ensure conservative inference, only predictors with *p* ≤ 0.01 were retained in the final models. Fully saturated models including all retained predictors were also constructed to estimate the total variability explained (DevSat). All statistical analyses were conducted using Statistica 12 package (StatSoft, Inc, 2014).

### Spatial autocorrelation analyses

Spatial autocorrelation in model residuals was analysed as evidence of structured deviations from environmentally driven expectations, in order to determine whether a structured spatial pattern remained in the data after accounting for the selected predictors (Diniz-Filho, Bini & Hawkins, 2003). To achieve this, local indicators of spatial association (LISA) were calculated for each grid cell (Anselin, 1988). Correlograms were generated for the first ten distance lags, with the first lag defined to include the eight cardinal directions (Carl & Kühn, 2007). LISA analyses were performed using SAM (Spatial Analysis in Macroecology; Rangel, Diniz-Filho & Bini, 2010; www.ecoevol.ufg.br/sam). Normality and homoscedasticity of residuals were also assessed following standard procedures (Crawley, 1993).

## Results

The negative exponential function adequately described the decline in the number of grid cells as survey effort increased across all spatial resolutions (Fig. 2), with strong negative correlations ranging from −0.74 to −0.99 (Table 2). Irrespective of spatial resolution, only a small fraction of cells could be considered well surveyed. Under the most stringent criterion, considering cells with numbers of navigation routes and travelled kilometres equal to or greater than the values corresponding to the 95th percentile of survey effort, an average of approximately 5.8% of all cells met this condition. When a less restrictive threshold was applied (90th percentile), the proportion of well-surveyed cells increased to approximately 12.3% (Table 2). Together, these patterns indicate a strong spatial concentration of survey effort across all spatial resolutions.

**Table 2:** Total number of grid cells in the Gulf of California (Tcells) at different spatial resolutions. Frequency distributions of the number of navigation routes and travelled kilometers per cell (expressed as ln +1) were fitted to a negative exponential function (Fig. 2), with *R^2^* indicating the coefficient of determination of the models. C10 and C5 denote the number of cells with numbers of navigation routes and travelled kilometres equal to or greater than the 90th and 95th percentiles, respectively. Nr10 and Nr5 represent the minimum numbers of navigation routes and travelled kilometers required for a cell to be classified within 90th and 95th percentiles. In all cases, the first value before the slash corresponds to navigation routes and the second to travelled kilometers.

Resolution	Tcells	*R* ^*2*^	C10	C5	Nr10	Nr5
5 × 5 km	13,787	0.96/0.98	2,192/2,027	813/721	≥2/9.9	≥3/14.8
10 × 10 km	2,290	0.96/0.96	299/264	131/115	≥3/31.2	≥5/62.4
20 × 20 km	726	0.92/0.99	85/81	50/37	≥3/27.1	≥4/44.5
30 × 30 km	229	0.86/0.96	23/23	16/12	≥6/157.1	≥8/301.0
50 × 50 km	91	0.74/0.91	13/10	6/5	≥8/602.2	≥13/1,264.8

GLMs revealed a clear scale-dependent pattern in the number of environmental predictors significantly associated with the two variable measuring survey effort. At the coarser resolutions (50 × 50 km and 30 × 30 km), between two and four variables were statistically significant. Notably, among the eight variables that were statistically significant for at least one of the two survey-effort surrogates, only primary productivity (PP) consistently appear in both, always showing a negative relationship (Table 3). Thus, higher productivity was associated with lower survey effort. At the intermediate resolution (20 × 20 km), five or six predictors were statistically significant depending on the survey-effort surrogate analysed, with phytoplankton concentration (PHY), primary productivity, and sea surface temperature (SST) showing significant relationships irrespective of the surrogate considered. In this case, greater survey effort was associated with lower productivity and temperature, but with higher phytoplankton concentration (Table 3). At finer resolutions (10 × 10 km and 5 × 5 km), almost all considered variables were statistically significant on at least one occasion. In particular, both survey-effort surrogates showed a consistent positive relationship with phytoplankton concentration and negative relationships with distance to the nearest populated locality (DP), primary productivity, and sea surface temperature (Table 3). Thus, the most intensively surveyed areas at finer resolutions tended to occur in colder waters with high phytoplankton concentration but lower primary productivity, and located close to populated terrestrial localities. Interestingly, the sign of the relationships with bathymetry (BAT) and pH (PH) was negative for the number of navigation routes, but positive for travelled kilometres (Table 3).

**Table 3:** Statistically significant predictors. Statistically significant predictors, as identified by Wald statistics, explaining whale survey effort in the Gulf of California expressed as number of navigation routes from 1987 to 2018 (A) and kilometres travelled (B) across the five spatial resolutions analysed (*p ≤ 0.01, **p ≤ 0.001, ***p ≤ 0.0001). Signs in parentheses indicate the direction of the relationship (positive or negative). Dev represents percentage of deviance explained by the significant predictors, whereas DevSat indicates the total deviance explained by all predictors included simultaneously (saturated models). Variable acronyms are as follows: chlorophyll *a* (CHLA), dissolved molecular oxygen (DMO), nearest populated locality (DP), slope (SLO), phytoplankton concentration (PHY), particulate organic carbon (POC), primary productivity (PP), sea surface temperature (SST), bathymetry (BAT), and pH (PH).

Resolution	CHLA	DMO	DP	SLO	PHY	POC	PP	SST	BAT	pH	Dev	DevSat
**A**												
**50 × 50 km**	-	-	-	-	10.04* (+)	-	9.15* (−)	8.62* (−)	9.42* (−)	-	58.82	75.38
**30 × 30 km**	-	9.23* (+)	-	-	-	7.09* (+)	-	29.94*** (−)	13.83** (−)	-	61.45	66.57
**20 × 20 km**	-	47.73*** (+)	-	-	32.32*** (+)	88.14*** (+)	32.32*** (−)	20.67*** (−)	18.42*** (−)	-	47.77	48.65
**10 × 10 km**	20.80*** (+)	-	99.55*** (−)	-	99.55*** (+)	11.16*** (+)	99.55*** (−)	130.90*** (−)	224.20*** (−)	224.20*** (−)	50.36	50.38
**5 × 5 km**	7.12* (+)	47.21*** (+)	308.53*** (−)	-	263.50*** (+)	51.67*** (+)	214.13*** (−)	290.66*** (−)	-	39.02*** (−)	30.77	30.79
**B**												
**50 × 50 km**	-	14.29** (+)	10.74* (−)	-	-	-	15.34** (−)	-	-	21.43*** (+)	74.04	75.99
**30 × 30 km**	-	-	80.70*** (−)	-	-	-	35.47*** (−)	-	-	-	63.33	65.15
**20 × 20 km**	-	-	54.51*** (−)	-	22.56*** (+)	–	114.50*** (−)	10.00* (−)	-	27.86*** (+)	39.75	40.29
**10 × 10 km**	-	57.62*** (+)	87.67*** (−)	22.29*** (+)	35.19*** (+)	6.73* (−)	192.58*** (−)	-	24.04*** (+)	137.95*** (+)	45.49	45.50
**5 × 5 km**	-		602.10** (−)	82.23*** (+)	31.21*** (+)	-	509.68*** (−)	76.29*** (−)	-	89.59*** (+)	21.87	21.89

**Figure 3:**
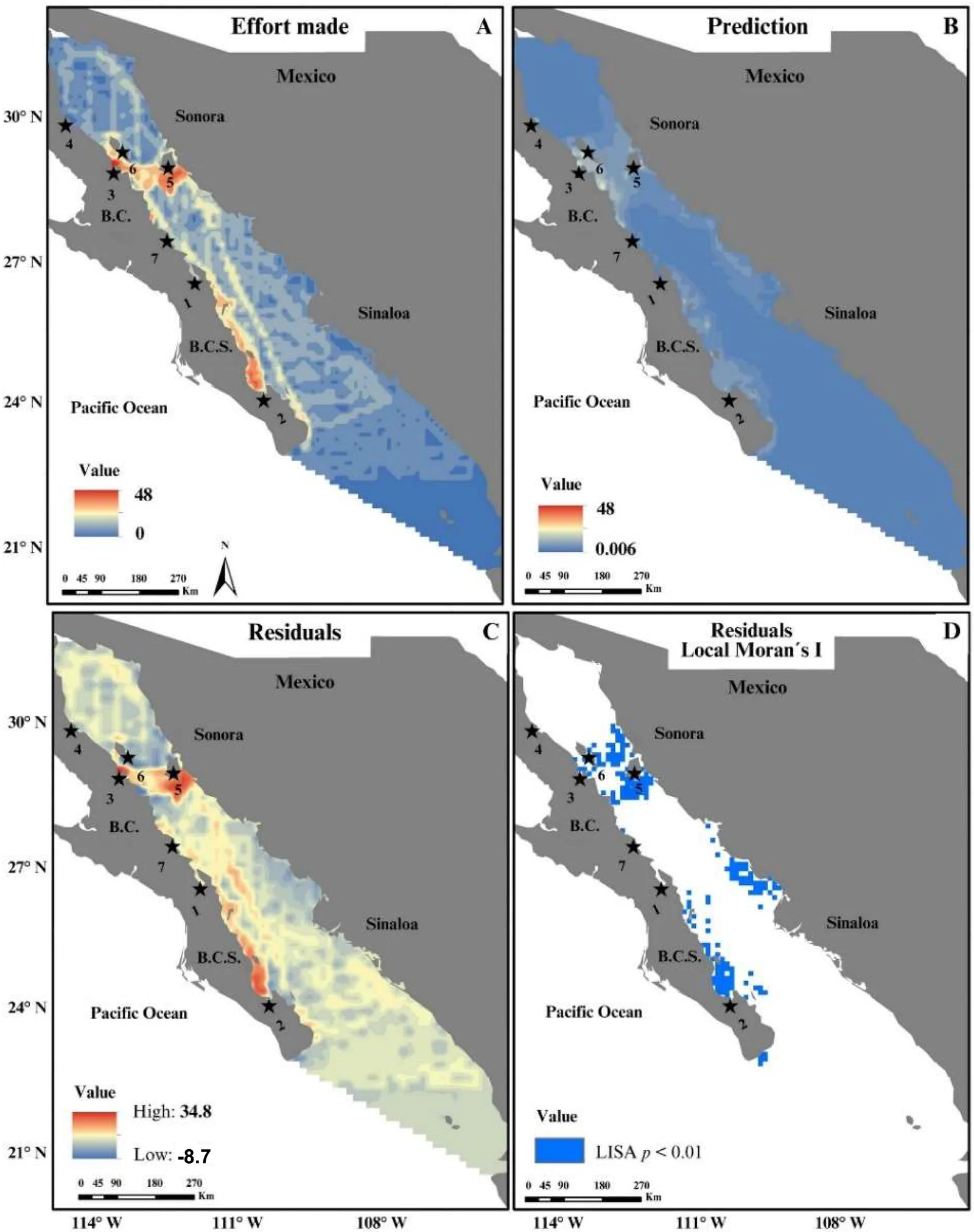
Maps derived from GLMs. (A) Map illustrating the spatial distribution of survey effort conducted by different organizations during whale-monitoring programmes in the Gulf of California. The scale indicates the number of times each grid cell was visited. Numbered locations correspond to: 1 = Bahía Concepción, 2 = Bahía de La Paz, 3 = Bahía de los Ángeles, 4 = Bufeo, 5 = Isla Tiburón, 6 = Isla Ángel de la Guarda, 7 = Santa Rosalía. (B) Predicted survey effort based on the model fitted using the selected environmental variables. (C) Spatial distribution of model residuals, with blue indicating overpredicted areas and red indicating underpredicted areas. (D) Results of the local indicator of spatial association (LISA) analysis based on the local Moran’s index, highlighting areas with statistically significant local spatial autocorrelation (blue). All maps are displayed at a spatial resolution of 10 × 10 km; however, the observed patterns were essentially identical regardless of the spatial resolution considered and the variable used as a surrogate of survey effort (number of navigation routes or travelled kilometres).

The residual structure of the models provides the strongest evidence for non-random, knowledge-driven allocation of survey effort. Maps of predicted and residual values (Figs. 3B and 3C) across all spatial resolutions revealed a systematic underestimation of survey effort in cells with the highest number of navigation routes. This spatial pattern was essentially identical when travelled kilometres were used as the response variable (not shown). A strong positive correlation existed between survey effort and the residuals of the saturated models including all environmental predictors (*r* = 0.797; *p* < 0. 001 for the number of navigation routes, and *r* = 0.957; *p* < 0. 001 for travelled kilometres). Furthermore, these relationships were independent of the spatial resolution considered (not shown). For example, correlation values between travelled kilometres and residuals of the saturated models ranged from 0.991 at 5 km resolution to 0.892 at 50 km resolution. Spatial autocorrelation analyses further reinforced this result (Fig. 3D). Specifically, areas surrounding Ángel de la Guarda Island, Tiburon Island, and Concepcion Bay were consistently underpredicted, indicating survey effort higher than expected based solely on environmental conditions. Conversely, the southern sector of La Paz Bay, the region between the southern coast of Sonora and the coast of Sinaloa, and the Grandes Islas area off Bahia de Los Ángeles were overpredicted, suggesting lower-than-expected survey effort in these areas (Figs. 3C and 3D).

## Discussion

In this study, we analysed the spatial distribution of whale survey effort in the Gulf of California to evaluate whether observed gaps in biological information primarily reflect insufficient sampling or, alternatively, the cumulative effects of prior knowledge guiding survey decisions. Our results demonstrate that only a small fraction of spatial units can be considered well surveyed, and that this pronounced spatial bias persists across all spatial resolutions analysed. Even under relatively permissive criteria, fewer than 10% of grid cells concentrated the highest levels of survey effort, indicating a strong spatial aggregation of data collection activities.

Such imbalances are consistent with patterns widely documented in biodiversity databases across a broad range of taxonomic groups and regions (*e.g.*, Boakes et al., 2010; Lobo et al., 2018; García-Roselló, González-Dacosta & Lobo, 2023). They underscore the limitations of opportunistic data collection for accurately characterising species distributions, particularly for highly mobile marine organisms, and reinforce the need for carefully designed survey strategies to obtain spatially representative datasets (Tyne et al., 2016; Mannocci et al., 2018). Data collection often occurs opportunistically and is based on prior knowledge of areas with high animal occurrence (Kot et al., 2010; Embling, Walters & Dolman, 2015; Tyne et al., 2016). In the Gulf of California, logistical constraints further exacerbate these biases. Most surveys are conducted from small vessels operating close to the coast, while offshore areas can only be accessed by a limited number of larger platforms or through aerial surveys, both of which involve substantially higher costs (Evans & Hammond, 2004). As a result, survey effort tends to concentrate in accessible areas where whale encounters are more likely, reinforcing pre-existing spatial biases in the available data.

Importantly, our results do not merely document uneven sampling, but reveal a directional process in which accumulated knowledge actively shapes the allocation of survey effort. This spatial heterogeneity raises a fundamental question: do poorly surveyed areas represent genuinely unexplored regions that may harbour high whale densities, or do they reflect locations that have been implicitly classified as biologically uninformative on the basis of accumulated knowledge? In other words, does the observed distribution of survey effort primarily arise from environmental and socio-economic constraints, or does it reflect a selective process driven by expectations of whale occurrence? Distinguishing between these alternatives is central to understanding whether current whale databases in the Gulf of California are better characterised as sampling-limited or knowledge-limited inventories. Our results provide strong evidence supporting the latter scenario. The spatial distribution of model residuals revealed systematic underestimation of survey effort in cells with the highest number of navigation routes or travelled kilometres, a pattern that persisted after accounting for a wide range of environmental and geographic predictors. The strong positive correlation between survey effort and model residuals, together with their significant spatial autocorrelation, indicates that certain areas have been surveyed far more intensively than expected based on environmental conditions alone. Importantly, many of these over-surveyed areas coincide with regions independently identified as hotspots of whale occurrence. For example, satellite-derived analyses have highlighted the waters between Bahía de los Ángeles and Ángel de la Guarda Island, as well as the route between Loreto and La Paz, as areas of high fin whale occurrence (Jiménez López et al., 2019). The northern Gulf of California has also been recognised as a key foraging area for fin whales exploiting surface swarms of euphausiids (Ladrón de Guevara, Lavaniegos & Heckel, 2008), while Bahía de La Paz supports a high diversity of cetacean species (Salvadeo et al., 2011; Pardo et al., 2013; Antichi et al., 2022). These independent lines of evidence strongly suggest that survey effort has been preferentially directed towards areas already known to support high whale densities.

Environmental variables explained only a limited proportion of the spatial variation in survey effort, indicating that environmental suitability alone does not determine where surveys are conducted. Nevertheless, as spatial resolution increased, a greater number of environmental predictors became statistically significant, although their overall explanatory power declined. This pattern probably reflects a scale-dependent shift in the processes underlying survey allocation. At coarse resolutions, broad environmental gradients dominate the spatial structure of survey effort, allowing a relatively small number of predictors to explain a large proportion of the observed variability. In contrast, at finer resolutions, local-scale heterogeneity increases substantially, and survey effort becomes influenced by a larger number of partially independent environmental, logistical, operational, and contingent factors. Consequently, more predictors become statistically detectable, but each explains only a small fraction of the total variability, thereby reducing the overall explanatory power of the models. This pattern may suggest that survey allocation is increasingly shaped by accumulated prior knowledge rather than by environmental suitability alone, and that this process is more readily detectable at finer spatial resolutions.

With respect to environmental control of survey effort irrespective of the spatial resolution and used survey effort surrogate, areas receiving the greatest survey effort were associated with colder waters, located near to populated localities, but having higher concentrations of phytoplankton and lower primary productivity. The negative relationship between primary productivity and phytoplankton concentration is common in nutrient-rich and upwelling zones (Vallina et al., 2014) where growth rates are high but residence times are short, because a lot of carbon is produced each day but zooplankton quickly consumes the chlorophyll production. These conditions are broadly consistent with known patterns of whale distribution in the Gulf of California (Pardo et al., 2013; García-Morales, Pérez-Lezama & Shirasago-Germán, 2017) and in other marine systems worldwide (Scales et al., 2017; Meynecke et al., 2021). However, such variables often exhibit limited predictive capacity when used in isolation (Higby, Stafford & Bertulli, 2012; Chavez-Rosales et al., 2022), particularly when analyses rely on surface sightings that may be influenced by dive behaviour, prey distribution at depth, or other unmeasured factors.

Taken together, these findings indicate that the spatial distribution of survey effort in the Gulf of California is more consistent with a knowledge-limited inventory than with a purely sampling-limited one. Survey effort appear to be disproportionately concentrated in areas where whales are already known to occur frequently, while environmentally suitable but less well-known regions remain comparatively under-sampled. This pattern likely reflects a feedback mechanism in which prior knowledge of whale distribution shapes survey design, which in turn reinforces existing spatial biases in the data. Such feedbacks are particularly likely in regions with long histories of research activity, where accumulated experience and unpublished knowledge influence decisions about where to allocate limited sampling resources. These results have important methodological implications. Firstly, under knowledge-limited scenarios, our results do not imply that poorly surveyed areas should automatically be interpreted as having low biological value, nor that survey effort should be ignored in distributional analyses. Instead, they suggest that researchers should first evaluate whether observed spatial gaps arise primarily from insufficient sampling or from historically knowledge-driven survey allocation. This distinction may be important because conventional corrections based solely on sampling effort may not be fully adequate when effort allocation is itself biologically structured. In such cases, poorly sampled regions may remain systematically underrepresented through time, limiting the ability to detect ecological change, range shifts, emerging hotspots, or previously unrecognised areas of biological importance. Secondly, knowledge-limited scenarios imply that correlative models used to predict species distributions or habitat suitability can be strongly affected by spatially biased sampling effort, especially when extrapolating into poorly surveyed areas (Elith & Leathwick, 2009; Chavez-Rosales et al., 2022). Even when opportunistic data are used, accounting explicitly for survey effort can improve model interpretation and reduce the risk of spurious inferences (Higby, Stafford & Bertulli, 2012; García-Roselló et al., 2015; Tang, Clark & Gelfand, 2021). Our findings highlight the need to consider the reciprocal relationship between organism distributions and data collection processes, particularly for highly mobile vertebrates such as cetaceans. Recognising when biodiversity data are shaped by knowledge-limited rather than sampling-limited processes is therefore essential to avoid reinforcing historical biases in biogeographical inference and conservation planning, and to distinguish true information gaps from areas that are under-sampled due to perceived low biological value. In conclusion, explicitly incorporating survey effort into spatial analyses can help prevent misinterpretations, guide more balanced sampling strategies, and ultimately improve the reliability of biodiversity assessments and conservation planning in marine systems.

## Conclusions

This study set out to test whether spatial gaps in whale records from the Gulf of California are better explained by a sampling-limited process, in which uneven data reflect insufficient coverage, or by a knowledge-limited process, in which accumulated experience and expectations shape where survey effort is allocated. Our results provide consistent support for the knowledge-limited hypothesis. Survey effort was extremely concentrated across all spatial resolutions, with fewer than 10% of grid cells accounting for the highest levels of sampling. Although several environmental and geographical predictors were significantly associated with survey effort, their explanatory power was limited. Crucially, residual and spatial autocorrelation analyses revealed systematic underestimation of effort in a small number of heavily surveyed areas. These areas coincided with independently identified hotspots of whale occurrence, indicating that survey effort have been disproportionately directed towards locations already known to support high whale densities. This pattern cannot be attributed solely to environmental suitability and instead reflects a structured, non-random allocation of effort consistent with a knowledge-limited inventory process.

The principal contribution of this study lies in explicitly distinguishing between sampling-limited and knowledge-limited mechanisms as alternative explanations for spatial bias in biodiversity databases. While uneven sampling is widely recognised, the possibility that prior knowledge actively reinforces spatial bias has rarely been formally evaluated. By integrating multi-scale spatial analyses, environmental modelling, and residual spatial structure, we demonstrate that survey effort and biological knowledge are reciprocally linked. This conceptual clarification has methodological implications for the interpretation of opportunistic datasets and for the design of cetacean monitoring strategies.

Several limitations should be acknowledged. First, navigation routes and travelled kilometres were used as a proxy for survey effort, which, although robust, may not fully capture variation in detectability, observer experience, or temporal heterogeneity. Second, the environmental predictors considered, while comprehensive, cannot represent all ecological processes influencing whale distribution, particularly subsurface prey dynamics. Finally, the study focuses on a single, intensively studied marine system; caution is therefore warranted when extrapolating these conclusions to other regions without comparable research histories. Future research should evaluate whether knowledge-limited dynamics occur in other taxonomic groups and marine regions with long-term monitoring programmes. Integrating independent platforms (*e.g.*, aerial surveys, passive acoustic monitoring, or systematic offshore transects) could help test whether under-surveyed areas truly harbour low whale densities or simply reflect historical neglect. Methodologically, incorporating explicit models of survey allocation decisions into species distribution modelling frameworks represents a promising direction.

## Supplemental Information

10.7717/peerj.21685/supp-1 Supplemental Information 1 Data. Database containing the number of navigation routes followed by tracking and research teams (ROCELL), as well as the kilometres travelled during surveys (KMCELL), for each grid cell at the five spatial resolutions used (5 x 5 km, 10 × 10 km, 20 × 20 km, 30 × 30 km, 50 × 50 km). Longitude and latitude represent the centroid of each cell. Relatively well-surveyed c ells, defined as those with numbers of navigations routes and travelled kilometres equal to or greater than the values corresponding to the 90th and 95th percentiles, were identified using binary P90 and P95 columns (1 = well-surveyed; 0 = otherwise).

## Additional Information and Declarations

### Data Availability

The following information was supplied regarding data availability:

The data is available in the Supplemental File.

Raw navigation tracks are available from Dra. Abril Romo Piñera. Head of the Departament of Research, Directorate of Interdisciplinary Research and Graduate Studies, Universidad Autónoma de Baja California Sur. E-mail: akromo@uabcs.mx. Researchers interested in accessing the raw data should contact Dra. Romo Piñera directly.
